# Cutaneous Melanoma with Tonsillar Metastasis: Treatment with Cryoablation

**DOI:** 10.15388/Amed.2021.29.1.5

**Published:** 2022-07-25

**Authors:** Audrius Dulskas, Mantas Trakymas, Jolita Gibavičienė, Vytautas Čepulis, Agnė Čižauskaitė, Narimantas E. Samalavičius

**Affiliations:** Department of Abdominal and General Surgery and Oncology, National Cancer Institute, 1 Santariskiu Str., Vilnius LT-08406, Lithuania; Department of Radiology, National Cancer Institute, 1 Santariskiu Str., LT-08406, Vilnius, Lithuania; Department of Head and Neck Surgery and Oncology, National Cancer Institute, 1 Santariskiu Str., LT-08406, Vilnius, Lithuania; Department of Head and Neck Surgery and Oncology, National Cancer Institute, 1 Santariskiu Str., LT-08406, Vilnius, Lithuania.; Breast surgery Department, Oncology Chemotherapy Clinic, Klaipeda University Hospital, Liepojos St. 41, LT-92288 Klaipeda, Lithuania; Department of Surgery, Klaipeda University Hospital, 41 Liepojos Str., Klaipeda LT-92288, Lithuania

**Keywords:** Tonsil metastasis, Cutaneous melanoma, Cryoablation, Tonsillectomy, Radiology

## Abstract

Metastasis of cutaneous melanoma to the oral cavity is a rare condition. Male patient with cutaneous melanoma metastasis to right tonsil 2 years after radical skin lesion resection was treated with surgery: tonsillectomy and later resection of soft palate were performed. Two years later the patient was diagnosed with progressive disease to right tonsil and soft palate. Rapid inoperable relapse was detected six months later. The patient underwent two procedures of palliative cryoablation of the metastasis. Postoperative course was uneventful. Patient died 7 months later due to progression of the disease.

Cryoablation alone or together with surgery may be a relatively safe option for treatment of inoperable disease of head and neck tumours.

## Santrauka.

Odos melanomos metastazė burnos ertmėje pasitaiko retai. Vyriškos lyties pacientui, sergančiam odos melanoma, metastazė išplito į dešiniąją tonzilę praėjus dvejiem metams po to, kai buvo atliktas radikalus odos pažeidimo pašalinimas atlikus chirurginę operaciją – tonzilektomiją, o vėliau pašalintas minkštasis gomurys. Praėjus dvejiem metams diagnozuota, kad paciento liga progresavo ir išplito į dešiniąją tonzilę ir minkštąjį gomurį. Po šešių mėnesių nustatytas staigus neoperuotinas atkrytis. Pacientui atliktos dvi metastazę lengvinančios krioabliacijos procedūros. Pooperacinė veikla buvo sklandi. Pacientas mirė praėjus 7 mėnesiams po gydymo dėl progresavusios ligos.

Krioabliacija, taikoma atskirai ar kartu su chirurginiu gydymu, gali būti gana saugus būdas gydyti neoperuotinus galvos ir kaklo auglius.

## Introduction

Melanoma accounts for less than 2% of skin cancer cases but causes a large majority of skin cancer deaths [1, 2]. Primary head and neck mucosal melanomas are rear accounting for 0.03% of all cancers, 1-4% of all melanomas, 50-60% of all mucosal melanomas and only 4% of sinonasal malignancies [3]. Anorectal melanoma is another very rare entity with very poor prognosis [4]. Metastasis to the oral cavity from cutaneous melanoma is very rare. And the prognosis is very poor because it means the widespread dissemination of the disease. The first case was reported in 1912 by *Schmidt* [5] and less than 30 cases can be found in the literature since [6].

Here we present a novel case of metastatic melanoma restricted to the palatine tonsil occurring 18 months after the removal of the primary cutaneous lesion. The patient underwent surgery, chemotherapy and radiotherapy. Unfortunately, the disease relapsed again with the metastasis to the same region – right soft palate. This time it was inoperable and it was decided to perform cryoablation of the metastasis. The patient underwent two procedures of cryoablation. The postoperative course was uneventful.

## Case Report

The case was reviewed and approved by the Institutional Review Board. Informed consent was taken from the patient.

A 53 years old male of body mass index of 27 kg/m2 without any comorbidities or pathological findings on laboratory testing on preoperative evaluation underwent local excision of changed 2 × 3 cm nevus of the right calf in November, 2011. The histopathological examination showed superficially spreading melanoma with tumour thickness 1.1 mm and Clark level IV. The margins were clear. There was no clinically or on radiological examination detectable lymphadenopathy. The patient did not undergo any further treatment.

18 months later he presented to ears nose and throat (ENT) clinic with a month history of sore throat, difficulty in swallowing, foreign body sensation. On examination dark pigmented exophytic with signs of bleeding mass was seen in the right palatine tonsil. Metastasis (MTS) of melanoma was confirmed by cytology. No other sites of MTS were found by the whole body computerized tomography (CT) scan. The patient underwent right tonsillectomy. Histopathological examination showed metastasis of cutaneous melanoma with BRAF wild-type. Resection margin was not assessed due to fragmentation of the specimen. The patient was further treated with 5 cycles of *Dacarbazine* (250 mg/square meter body surface/day IV for 5 days, every 3 weeks).

Six months later the patient presented to our clinic again with the same symptoms. Magnetic resonance imaging (MRI) scan of the head and neck revealed 14 × 8.4 mm mass in the right part of palatoglossal arch and right palatine tonsil. Patient underwent extirpation of right soft palate tumour. Histopathology examination showed ulcerated MTS of melanoma with nonassessed margin because of fragmentation. Subsequently the patient was prescribed for external radiotherapy. The daily dose of 2 Grays (Gy) was given in 15 fractions, with total dose of 30.0 Gy to the right tonsillar fossa and regional lymph nodes. He tolerated radiotherapy well and no treatment-related side effects were observed. Normal swallowing function was established. For palliative care *Interferon α* was then prescribed (200 mg twice a day). The patient was on periodical follow-up.

In August, 2014 the 26 mm tumour of left kidney was verified to be grade 2 clear cell renocellular carcinoma. The thermoablation of this tumour was planned but imaging of head and neck showed tumour progression at the right tonsillar area. Resection was performed in March, 2015. Histopathology again reported the MTS spread with clear resection margins.

**Figure 1.**
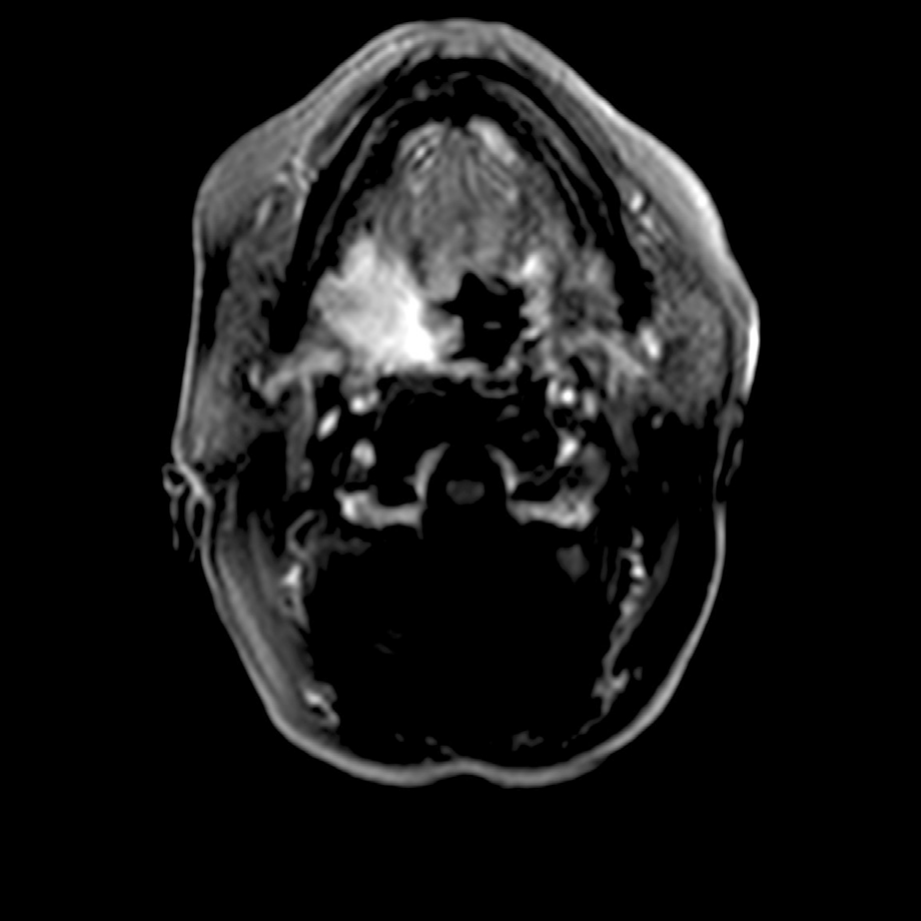
Contrast enhanced magnetic resonance imaging (MRI) before cryoablation – 10 cm in length tumour invading parapharyngeal, submandibular, palatoglossal arch and palatopharyngeus.

**Figures 2–4.**
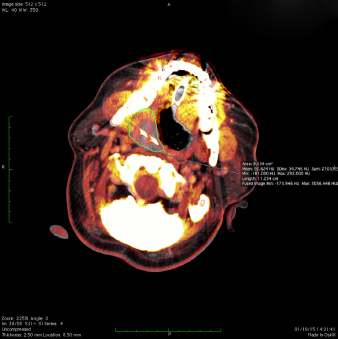

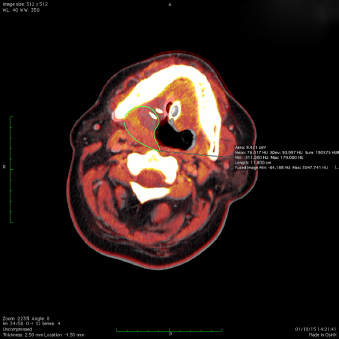

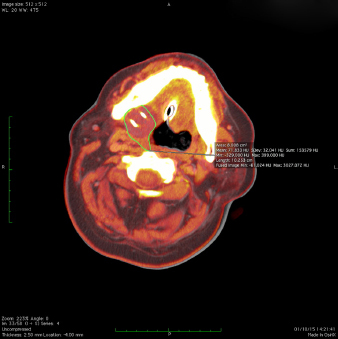
Fusion of the preablation magnetic resonance imaging (MRI) and the ablation computed tomography (CT) volume during cryoablation. Three different positions of cryoprobes and ice balls encompassing the MRI visible tumour volume (green line).

**Figure 5.**
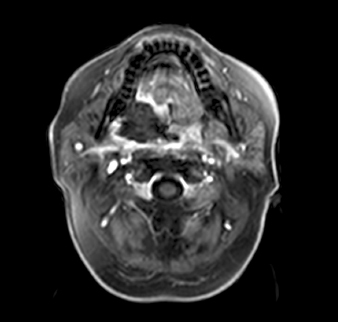
Postablation contrast enhanced MRI image, ablation zone encompassing the tumour.

In October, 2015 around 10 cm in length tumour of irregular form invading parapharyngeal, submandibular, palatoglossal arch, soft palate and palatopharyngeus was seen on the MRI scan of head and neck (Figure 1). The main mass was in the right tonsillar sinus with signs of mandible infiltration. Due to infiltration to other vital structures (nerves and vessels) the tumour was considered to be inoperable but the rapid exophytic growth with obstructive symptoms required some intervention. Multidisciplinary team decided to start treatment with palliative cryoablation to reduce tumour burden and chemotherapy thereafter. Two 17 gauge applicators (Ice Sphere, Galil Medical Inc.) were used. Ablation was performed under ultrasound, CT and fusion CT/MRI guidance (Figures 2–4). When applicators were in place, two standard 10 + 10 min freeze and thaw cycles were initiated. Irregular configuration of the tumour and its size made a radiologist change the position of applicators to three different locations to ensure the whole tumour mass to be treated. Postprocedural diagnostic CT scan was performed to assess the ablative effect. Large area of necrosis was seen on the follow up CT scan of head and neck encompassing the whole tumour area with no ablative margin near vital structures. As there was local tumour progression detected seven weeks later on the control MRI scan of the head and neck, the resection of exophytic compound of the tumour was performed and cryoablation of tumours in soft head and neck tissues was repeated two weeks later (Figure 5). No complications were seen and the patient was discharged on day 5. The patient had no neurological or functional deficit, vascular complication, or adverse cosmetic sequelae. Chemotherapy with carboplatinum and paclitaxel was started one month later. Nonetheless the patient died seven months later due to rapid progression of the disease.

## Discussion

Cryosurgery is the therapeutic use of cold to induce tissue necrosis with ablative intent. The first report of the use of local freezing as a treatment modality is attributed to *J. Arnott*, who described in 1850 the direct application of a salt-ice mixture to various skin lesions [7]. He noticed a marked anaesthetic and haemostatic effect and advocated its use, mostly as a palliative treatment, in a large variety of diseases. Solid carbon dioxide, cold air blast, and liquid nitrogen (LN) were used as cryogenic agents in the treatment of various benign and malignant lesions and achieved good results in terms of local tumour control and residual scarring [8]. In 1962 *Cooper* described a cryotherapy unit in which LN was circulated through a hollow metal probe [9]. In the last decade cryoablation is widely used in oncology for treatment purposes. We used this treatment technique for the patient with inoperable cutaneous melanoma metastasis to tonsil. The patient underwent 2 procedures of cryoablation with acceptable results. The patient had no complications, and there was a great reduction of tumour mass. If the tumour would be smaller, image-guided ablative treatment could give even better results as the ablation zone could encompass even infiltrated bony or other structures, where palliative surgery would be too traumatic. Ablative treatment also does not affect larger blood vessels that are very important in head and neck area.

Only one single centre retrospective cohort study on cryoblation as a treatment of head and neck tumours can be found in the literature [10]. Authors report nine cases of patients undergoing cryoablation as a salvage surgery. The successful tumour control with good functional outcome was reached in eight patients.

Metastatic melanoma in the palatine tonsil is very rare [6]. There is no tropism between left or right tonsil and any objective tendency for bilateral involvement [11]. But, a male predominance may be real. Average age of affected patients was 44 years. In previous case reports, the interval between excision of the primary lesion and metastasis to the palatine tonsil ranged from 4 to 84 months (mean 32), compared with 18 months in our patient. The palatine tonsils are secondary lymphoid organs containing aggregations of lymphoid cells but, unlike lymph nodes, they do not possess afferent lymphatics. Therefore, tonsillar metastasis is haematogenously disseminated, implying a poor prognosis. It is clear evidence of dissemination and should lead to an immediate extensive work-up. The most common presenting symptoms of tonsil metastasis are throat soreness and/or dysphagia. Odynophagia, bleeding and ear pain may occur in late stages. The lesion may be symptomless [12]. Our patient was complaining of throat sore, difficulty in swallowing and foreign body sensation. Clinically, it appears as a brown or dark pigmented tonsillar lesion in a patient with a previous history of malignant melanoma. *Henderson et al.* reported 16 patients who had melanoma with metastasis to the tonsil who died soon afterwards, despite various therapies [13]. Our patient has remained disease-free for more than 18 months since the excision of the palatine tonsil. The treatment of tonsillar metastases is mainly palliative due to the disseminating nature of the underlying disease and the advanced tumour stage of patients with such metastases. Wide tonsillectomy provides local comfort and a specimen for histology. Supportive therapy includes management of other symptomatic metastasis.

However, the systemic treatment of metastatic mucosal melanoma has changed since 2010. At present, for all the patients with advanced cutaneous melanoma the mutation at the V600 site in BRAF status should be assessed. Patients with resected stage IV melanoma should be offered adjuvant nivolumab [14]. Patients with resected stage IV melanoma may be offered pembrolizumab or (in the case of BRAF-mutant disease) dabrafenib plus trametinib. For patients with BRAF wild-type unresectable/metastatic cutaneous melanoma, the following treatment options should be offered (in no particular order): ipilimumab plus nivolumab followed by nivolumab or nivolumab or pembrolizumab, immunotherapy with nivolumab in combination with ipilimumab is recommended [14, 15]. After progression on anti-PD1 therapy, patients with unresectable/metastatic BRAF wild-type cutaneous melanoma may be offered ipilimumab or ipilimumab-containing regimens.

New target therapy drugs may be used for metastatic disease in the clinical setting as well. Unfortunately, these drugs are not freely reached or entirely reimbursed in our country.

Obviously, paper is limited by the reporting only single case, without possibility to examine our technique in a larger cohort.

In conclusion, metastatic cutaneous tonsillar disease is a very rare entity with a poor prognosis. Cryoablation alone or together with surgery may be a relatively safe option for treatment of an inoperable disease of head and neck tumours for normal function preservation. Further larger cohort studies are needed to affirm the safety and feasibility of this procedure.
